# Event-Related Potentials during a Gambling Task in Young Adults with Attention-Deficit/Hyperactivity Disorder

**DOI:** 10.3389/fnhum.2018.00079

**Published:** 2018-02-27

**Authors:** Sarah K. Mesrobian, Alessandro E. P. Villa, Michel Bader, Lorenz Götte, Alessandra Lintas

**Affiliations:** ^1^Neuroheuristic Research Group, University of Lausanne, Lausanne, Switzerland; ^2^LABEX, Faculty of Business and Economics, University of Lausanne, Lausanne, Switzerland; ^3^Research Unit of the University Department of Child and Adolescent Psychiatry (SUPEA), CHUV University Hospital and Faculty of Biology and Medicine, University of Lausanne, Lausanne, Switzerland; ^4^Institute for Applied Microeconomics and Bonn Graduate School of Economics of the University Bonn, Bonn, Germany

**Keywords:** ADHD, decision-making, evoked potentials, N2-P3, N400-like, personality

## Abstract

Attention-deficit hyperactivity disorder (ADHD) is characterized by deficits in executive functions and decision making during childhood and adolescence. Contradictory results exist whether altered event-related potentials (ERPs) in adults are associated with the tendency of ADHD patients toward risky behavior. Clinically diagnosed ADHD patients (*n* = 18) and healthy controls (*n* = 18), aged between 18 and 29 (median 22 Yo), were screened with the Conners' Adult ADHD Rating Scales and assessed by the Mini-International Neuropsychiatric Interview, adult ADHD Self-Report Scale, and by the 60-item HEXACO Personality Inventory. The characteristic personality traits of ADHD patients were the high level of impulsiveness associated with lower values of agreeableness. All participants performed a probability gambling task (PGT) with two frequencies of the feedback information of the outcome. For each trial, ERPs were triggered by the self-paced trial onset and by the gamble selection. After trial onset, N2-P3a ERP component associated with the attentional load peaked earlier in the ADHD group than in controls. An N500 component related to the feedback frequency condition after trial onset and an N400-like component after gamble selection suggest a large affective stake of the decision making and an emphasized post-decisional evaluation of the choice made by the ADHD participants. By combining ERPs, related to the emotions associated with the feedback frequency condition, and behavioral analyses during completion of PGT, this study provides new findings on the neural dynamics that differentiate controls and young ADHD adults. In the patients' group, we raise the hypothesis that the activity of frontocentral and centroparietal neural circuits drive the decision-making processes dictated by an impaired cognitive workload followed by the build-up of large emotional feelings generated by the conflict toward the outcome of the gambling choice. Our results can be used for new investigations aimed at studying the fine spatiotemporal distribution of cortical activity, and the neural circuits that underly the generation of that activity, associated with the behavioral deficits characteristic of ADHD.

## Introduction

Decision making is an essential phase in a volitional act, that follows problem analysis, deliberation and evokes execution. The processes involved in the cognitive control of action and thought operations are divided into two modes according to the model developed by Norman and Shallice (1986): (i) potentially demanding but routine actions or thought operations, when selected by well-learned triggered procedures, are sufficient to carry out a task satisfactorily; (ii) routine operations are insufficient to achieve the goal and some form of explicit modulation or novel activity must be carried out. This model incorporates two assumptions: the first is that on-line cognitive operations are processed through a large set of neuronal assemblies; the second is that mutual inhibitory networks exist for selecting which action is to be carried out when there are conflicts or competing possibilities. Executive functions are high-level cognitive abilities allowing individuals to optimize their decision-making competences (Guilford, 1972; Stuss, 1992; Jurado and Rosselli, 2007) (the Supervisory System of the Norman-Shallice model) coming into effective operation in the decision-making task whenever the routine at lower levels of the system cannot solve the conflict they have been presented (Del Missier et al., 2012). The integrity of a monitoring system capable, on the short-term, to compare the actual and expected outcomes and, on the long-term, to build-up and maintain the repertoire of response alternatives is necessary to achieve a correct decision-making process (Miyake et al., 2000). Behavioral determinants such as vigilance, motivation, and emotions exert a powerful influence on the overall cognitive framework of decision making (Coricelli et al., 2007; Reckless et al., 2013) and evidence exists that cognitive inhibition and switching are executive functions that mediate creative potential and intelligence (Nusbaum and Silvia, 2011; Benedek et al., 2012). Hence, decision making requires the ability to evaluate external demands and internal goals, to perform a value-based action selection among several alternatives depending on the perceived or estimated costs and benefits of each choice translated into an expected reward to the final choice (Medin and Bazerman, 1999; Hinson et al., 2003; Dickhaut et al., 2009).

Impaired decision making is among the characteristic symptoms of patients affected by Attention Deficit/Hyperactivity Disorder (ADHD). This behavioral disorder of childhood and adolescence is characterized by primary deficits of executive functions and clinical symptoms including excessive inattention, hyperactivity and impulsiveness that persist into adulthood in a vast proportion of the diagnosed adolescents (Barkley, 2002; Kessler et al., 2005b; Willcutt et al., 2005; Pierrehumbert et al., 2006; Mowinckel et al., 2015). Adults receive ADHD diagnoses in roughly equal proportions. No significant gender differences were observed in anatomical brain studies (Yang et al., 2008) of ADHD patients, despite ADHD is diagnosed more often in boys than in girls (Biederman et al., 2004) and a slight prevalence of females was reported in the gender distribution of young adults (Simon et al., 2009). Adult ADHD patients tend to shift their actions toward oppositional conducts affecting their social lives (Barkley et al., 1996; Barkley and Fischer, 2010; Spencer et al., 2014) including alcohol or drugs abuse (Lee et al., 2011; Biederman et al., 2012). Their difficulties in making decisions lead ADHD patients to choose riskier options with unfavorable outcomes in economic and financial settings (Barkley and Fischer, 2010; Mäntylä et al., 2012; Matthies et al., 2012). In addition to cognitive impairments, these patients exhibit affective and motivational deficits with an independent effect on their social problems (Sonuga-Barke, 2005; Retz et al., 2012). ADHD patients were characterized by specific personality traits based on the “Big Five” and HEXACO models with lower scores on conscientiousness, emotionality, and agreeableness (Goldberg, 1993; Gomez and Corr, 2014). In fact, when it comes to financial risk taking, only the traits of honesty-humility, emotionality, and conscientiousness appear to be associated with risky decision making (Weller and Thulin, 2012). Failure to learn from emotionally negative feedback is one of the characteristics of impulsive individuals, thus leading to choices in favor of immediate gains and problem gambling in ADHD adults (Groen et al., 2013; Dai et al., 2016).

Brain activity triggered in time by a repeating physical or mental stimulus recorded using electroencephalographic techniques and averaged over many repetitions of the same stimulus constitutes the basis of the event-related potentials (ERPs) (Picton et al., 2000). In children diagnosed with ADHD most investigations were focused on cognitive control, in particular visual attention (Karayanidis et al., 2000; Perchet et al., 2001), response inhibition (Liotti et al., 2005; Albrecht et al., 2008; McLoughlin et al., 2009) and error monitoring (Wiersema et al., 2005; van Meel et al., 2007). Abnormal cognitive ERP components were reported in ADHD patients tested with visual and auditory cues (Barry et al., 2003). However, differences in protocols and in patients' selection with respect to comorbid disorders raised controversial issues about the significance of ERP differences between healthy individuals and ADHD patients (Johnstone et al., 2013; Spronk et al., 2013; Gong et al., 2014). Even in presence of a behavioral performance that was not impaired (MacLaren et al., 2007) it is likely that early sensory processing is altered in ADHD patients, as suggested by reduced P1 and N2 and enhanced P2 components evoked by non-target stimuli, accompanied by changes in response inhibition associated with altered N2-P3 components (Bekker et al., 2005; Groom et al., 2008; Barry et al., 2009; Dhar et al., 2010; McLoughlin et al., 2010; Johnstone et al., 2013; Lenartowicz and Loo, 2014). The outcome evaluation in decision making under risk elicit late ERP components measured with latencies typically in the [400-900] ms range from the triggering event, referred to as N400-like and N500 for negative waves followed by LPP for late positive potential. These waves were less studied in ADHD, but they are relevant to decision-making processes under uncertainty. Following the appearance of the two initial cards in a blackjack game, an N500 wave over the frontal areas, characterized by a larger amplitude for losses compared to gains, was observed with the option to ask for another card or not (Polezzi et al., 2010). Large negative amplitudes for N500 were elicited by trials with a high conflict vs. trials with a low conflict (Yang et al., 2007). LPP latencies and amplitudes were affected by high reports of affective experience like comparing emotional to neutral pictures and when performing emotionally congruous relative to incongruous actions (Schupp et al., 2004; Dennis and Hajcak, 2009; Broyd et al., 2012; Bamford et al., 2015).

The aim of this study is to investigate which are the behavioral and neural correlates of risky gambling decision-making in young adults with deficits of executive functions associated with ADHD and personality assessed with the HEXACO Personality Inventory. To the best of our knowledge, this is the first study designed with this aim. Moreover, we introduce a new spatiotemporal conditional analysis taking into account the low (LF) and the high frequency (HF) conditions for each group of participants at each recording site. Our hypothesis is that young ADHD patients are characterized by abnormal dynamics while performing decision-making tasks and that specific ERP components characterize those patients. We suppose that difference between ADHD and controls appear at first in frontal areas when the cognitive load is associated with the stimulus contextual value, then followed by the build-up of an emotional response associated with the difficulty to evaluate the outcome of decision making. To test this hypothesis, we recorded behavioral and ERP data in a sample of young adults affected by ADHD during completion of a Probabilistic Gambling Task (PGT), where the participants had to choose the portion of a preassigned amount they had to gamble in a game. We have manipulated the frequency of the feedback information aiming to assess the ERP components associated with the anticipatory processing of the target stimulus, attentional priming, cognitive workload, and response selection and reprogramming.

## Methods and materials

### Statistical analyses

Statistical analyses were performed with the R language and environment for statistical computing (Venables and Ripley, 2002; R Core Team, 2013). For most variables, we report the median and the mean ± SEM. All statistical hypotheses were tested with a level of significance of *p* = 0.05, unless otherwise reported. For parametric comparisons of distributions, we used Student's *t*-test and Cohen's *d* effect size. For Chi-square tests, the effect size is reported by Φ for 2 x 2 contingency tables, otherwise by Cramer's *V* (Liebetrau, 1983). Non-parametric comparisons of sample distributions (Zeileis et al., 2008) were assessed by the Wilcoxon signed-rank test using the *Z* statistic for paired observations and by the Mann-Whitney *U* test for independent samples with effect size *r*. We have considered also large integrative models. Notice that most commonly used linear models are “fixed-effects-only” models assuming one or more fixed effects for each factor and a general error term. Instead of only one general error term, mixed-effects models add one or more random effects, independently for each factor, to the fixed effects. We used this class of models because we could not exclude a priori that within-subject factors are characterized by independent random effects. We used robust statistics throughout all the analyses (Boudt et al., 2012; Bodenhofer et al., 2013; Wang et al., 2014; Rousseeuw et al., 2015), including the robust correlation indexes ${\hat{\rho }}_{G}$, the robust mixed-effects model (Koller, 2016) and otherwise stated the linear mixed-effects models for within-subject factorial analyses (Bates et al., 2015).

### Participants' demography and assessment

This study was carried out in accordance with the recommendations of ethical and data security guidelines of the University of Lausanne with written informed consent from all subjects in accordance with the latest version of the Declaration of Helsinki (World Medical Association, 2013). The protocol was approved by the Cantonal Ethics Committee of the Canton Vaud (Switzerland). Ninetysix clinically referred young adult ADHD patients, aged between 18 and 29 years, were recruited either in the Psychiatric Department of the University Hospital of Lausanne or at a psychiatrist's practice in collaboration with the University Hospital after an initial screening appointment to ensure that they were fulfilling the criteria defined by the DSM-IV-TR for inattentive, hyperactive/impulsive or mixed subtypes (American Psychiatric Association, 2000). Patients with presence of motor tics, suicidal behavior, chronic medical conditions, and drug or alcohol abuse or comorbidity of psychiatric disorders, i.e., acute mood/anxious disorder, bipolar disorder, psychosis, autism or Asperger's syndrome, an antecedent of Tourette's syndrome, were excluded from this study. Patients taking psychostimulants were required to stop medication 24 h prior to testing. All patients taking any other psychotropic agents such as anti-depressants, mood stabilizers, non-stimulant medications for ADHD, or dopamine receptor-blocking agents were also excluded from this study. The slight prevalence of females in the gender distribution was in agreement with other reports (Simon et al., 2009). Control subjects were recruited among young adults matching the same age and gender of the patients, with an ideal target near 22 years old. Table 1 shows the main descriptive statistics of patients' demographics and behavioral assessment. In order to recruit controls, a call for participation was explicitly posted at the higher education institutions of the Lausanne-Geneva area, as well as at regional schools involved in vocational education, but apprentices and workers were included in the control sample. Notice that students from faculties of Economics and Psychology were a priori excluded from this study. All controls who expressed an interest in participating in the study were screened to ensure that they do not report any neuropsychiatric disorders or any other major chronic medical conditions and none were taking any psychoactive medications.

**Table 1:** Descriptive statistics (median, mean, and SEM) of participants' demographics, DSM-IV ADHD Symptom subscales, and personality traits.

	**Controls**	**ADHD**	**Between groups**	**Effect size**
N	18	18		
Age	22	22	*U* = 175	*r* = 0.07
	22.0 (0.8)	22.3 (0.7)	*p* = 0.69	
Female/Male	10/8	11/7	χ^2^ = 0.114	Φ = 0.06
			*p* = 0.74	
Laterality quotient (EHI)	95	100	*U* = 171.5	*r* = 0.05
	89.0 (5.5)	89.4 (5.5)	*p* = 0.76	
**CONNER'S ADULT ADHD RATING SCALES-SELF REPORT (SCREENING VERSION)**				
*CAARS-S:SV(T-score)*				
DSM-IV inattentive symptoms	52.5	79.5	*U* = 323^***^	*r* = 0.85
	51.7 (2.2)	79.6 (1.4)	*p* < 0.001	
DSM-IV hyperactive-impulsive	44.5	63.5	*U* = 304.5^***^	*r* = 0.75
Symptoms	43.9 (2.2)	64.8 (2.6)	*p* < 0.001	
DSM-IV total ADHD symptoms	50.0	77.0	*U* = 323^***^	*r* = 0.85
	47.9 (2.3)	76.5 (2.0)	*p* < 0.001	
ADHD Index	46.0	68.0	*U* = 324^***^	*r* = 0.86
	44.9 (1.4)	69.3 (1.3)	*p* < 0.001	
Adult ADHD self-report	48.0	67.0	*U* = 312^***^	*r* = 0.79
Scale(ASRS)	45.7 (2.2)	66.3 (1.9)	*p* < 0.001	
**HEXACO PERSONALITY FACTORS**				
[*H*]Honesty-humility	35.5	37.0	*U* = 150.5	*r* = 0.07
	36.5 (1.5)	35.1 (1.5)	*p* = 0.72	
[*E*]Emotionality	33.5	34.0	*U* = 165	*r* = 0.02
	32.7 (1.6)	32.3 (2.1)	*p* = 0.93	
[*X*]Extraversion	37.0	30.5	*U* = 87.5^*^	*r* = 0.39
	36.4 (1.1)	31.2 (1.7)	*p* < 0.05	
[*A*]Agreeableness	30.5	27.5	*U* = 96.5^*^	*r* = 0.35
	31.8 (1.2)	27.9 (1.5)	*p* < 0.05	
[*C*]Conscientiousness	35.0	28.5	*U* = 65.5^**^	*r* = 0.51
	35.4 (1.0)	29.6 (1.5)	*p* < 0.01	
[*O*]Openness to experience	31.0	38.0	*U* = 220.5	*r* = 0.31
	33.0 (1.6)	37.1 (1.6)	*p* = 0.06	

Two-sided p-value for group comparison and effect size are reported.

* p < 0.05;

** p < 0.01;

*** *p < 0.001*.

Two weeks prior the experimental session, all potential participants were requested to fill the Conners' Adult ADHD Rating Scales-Self Report (Screening Version, CAARS-S:SV) (Conners et al., 1999; Fumeaux et al., 2016) and the adult ADHD Self-Report Scale (ASRS) (Kessler et al., 2005a). The CAARS-S:SV includes the ADHD Index, referred to as CAARS in the text, the DSM-IV Inattentive Symptoms Subscale (CAARS-A), the DSM-IV Hyperactive-Impulsive Symptoms Subscale (CAARS-B) and the DSM-IV Total ADHD Symptoms Subscale (CAARS-C). CAARS was used because of its robust psychometric statistics and content validity in comparison to other scales (Taylor et al., 2011). A normalized *T*-score of CAARS > 60 for the ADHD group and a *T*-score of CAARS < 56 for the control group were set as inclusion criteria. Given these criteria, the final ADHD sample included 18 ADHD patients. After all screening procedures and inclusion criteria, we considered control subjects until matching the same sample size, i.e., 18 participants in the control group. Notice that in both groups CAARS and ASRS's total scores were positively correlated [i.e., ${\hat{\rho }}_{G}$(16) = 0.513, *p* = 0.03 for controls and ${\hat{\rho }}_{G}$(16) = 0.464, *p* = 0.05 for ADHD participants]. All 36 participants had a normal or corrected-to-normal vision, none reported a history of sustained head injury. We considered a possible bias associated with gender, but the robust statistics did not show any significant gender effect neither for CAARS [*F*_(1, 34)_ = 0.265, *p* = 0.61] nor for ASRS [*F*_(1, 34)_ 0.502, *p* = 0.48].

On the day of the experimental session, the participants were welcomed, then requested to complete the Edinburgh Handedness Inventory (EHI) (Oldfield, 1971) and underwent the Mini-International Neuropsychiatric Interview (MINI) (Sheehan et al., 1998) under the supervision of a trained clinical psychologist. All participants were also requested to answer the 60-item HEXACO Personality Inventory (Ashton and Lee, 2009). All items of HEXACO-60 employ a 1–5 response scale: 1 [*strongly disagree*] to 5 [*strongly agree*]. The HEXACO dimensions extraversion, conscientiousness and openness are comparable with the classical “Big Five” (BF) model of personality (Lee et al., 2005). However, the HEXACO dimensions agreeableness and emotionality are not identical to agreeableness and neuroticism dimensions of the BF. The major difference between the two models is the emergence of the honesty-humility dimension, which lies beyond the space of the BF (Goldberg, 1990; Costa Jr and McCrae, 1992; Ashton and Lee, 2005).

### Probabilistic gambling task (PGT) and behavior

**Figure 1:**
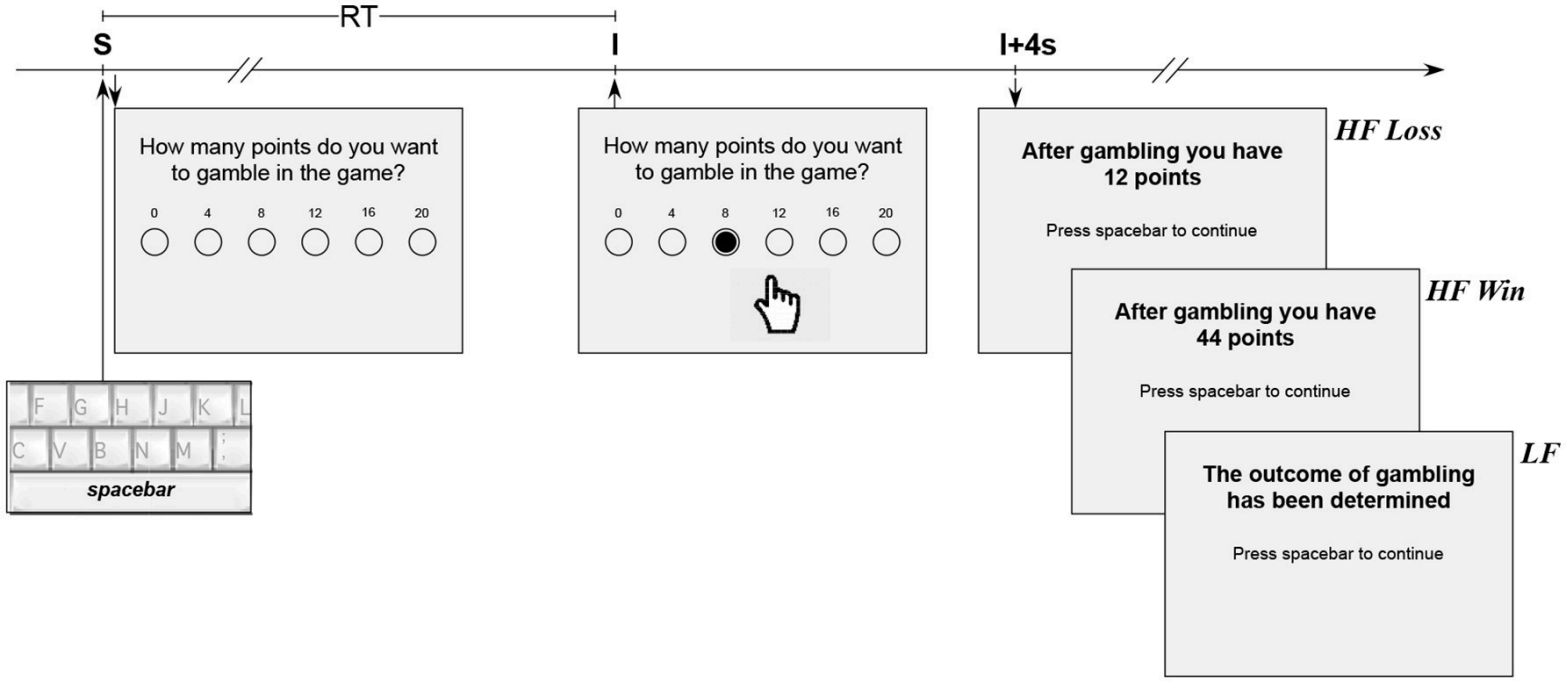
Experimental design of the probabilistic gambling task. Each trial began by pressing the spacebar (trigger event *S*), which was immediately followed by a message on the computer-controlled monitor with the request to choose a selected amount of points to gamble in a game. The response time (*RT*) was determined by the lag until the selection of the investment to gamble (trigger event *I*). After an additional fixed interval of 4 s (I+4s), the participant was informed about the outcome of the gamble (*HF Loss* or *HF Win*) in the *HF* condition or simply informed about the determination of the gamble in the *LF* condition.

The PGT is a modified version of the original Gneezy-Potters' task (Gneezy and Potters, 1997) described in detail elsewhere (Mesrobian, 2015). Each participant was endowed with an amount of 20 points at the beginning of each trial. The participants had to select an amount of points (among the values of 0, 4, 8, 12, 16, 20 points) to gamble in a trial (as illustrated by Figure 1). The graphical message with a grid corresponding to the possible choices was displayed on the computer-controlled monitor and the participant used the computer mouse to click on the selected amount of points to gamble. In order to reduce the saccadic eye movements, the graphical message was displayed in a screen area corresponding to a vertical angle of 3 degrees and a horizontal angle of 8 degrees, hence falling within the range of the normal human parafoveal region in reading (Rayner, 1998). Two events, trial onset (trigger event *S*) and gambling choice (trigger event *I*), delimited a time interval, the termination of which corresponds to a voluntary action, i.e., the choice of a selected amount to gamble. The outcome of the gambling was either to win four times the selected value, with a probability *P*_*win*_ = 1/3, or to loose the entire amount with a probability *P*_*loose*_ = 2/3 with a uniformly distributed probability (e.g., if the participant selected 8 points, the outcome would be 12 = (20−8) in case of loss, or 44 = (20−8) + (8 × 4) in case of win). For each trial in the “high-frequency feedback” condition (*HF*) the participant was informed, 4 s after the choice, about the amount of points held after gambling. In the “low-frequency feedback” condition (*LF*) the participant was just informed that the outcome of the gambling was determined. In both conditions (i.e., *HF* and *LF*) the overall amount of points held by the participant was displayed every four trials. Each participant played the PGT in 10 alternated blocks of *HF* and *LF* 16 trials each, hence 80 trials for each condition.

Participants' performance was assessed by the total gains earned after the end of playing the whole task (*TotG*), by a risk index (*RI*) and by response times (*RT*). In particular, we considered the total gains earned during low-frequency feedback trials (*TG*(*LF*)), and during high-frequency feedback trials (*TG*(*HF*)). The relative number of trials a participant gambled 0, 4, or 8 points defined a low-risk index *LR*. A high-risk index *HR* was defined as gambling amounts of 12, 16, or 20 points. A risk index *RI* centralized within the range [−1, +1] was calculated as *RI* = (*HR*−*LR*)/(*HR*+*LR*). Then, an *RI* toward −1 is characteristic of a risk-averse strategy, an index toward +1 for a risk-seeking participant, and *RI* ≈ 0 being associated with a risk-neutral attitude. Each participant could be further characterized with the corresponding *RI*s calculated following the feedback frequency, i.e., *RI*(*LF*) and *RI*(*HF*). The behavior of the participants was also assessed by measuring the response times (*RT*) in ms. The trials with *RT* < 250 ms and *RT* > 10 s were discarded. Additional trials detected as outliers on the basis of a robust analysis (Breunig et al., 2000) were also discarded from further analyses.

### EEG recording and analyses

Upon completion of the MINI, all participants included in the study were guided to a sound and light-attenuated room for the preparation of the EEG recordings. Electrophysiological signals were recorded using 64 scalp Ag/AgCl active electrodes (ActiveTwo MARK II Biosemi EEG System, BioSemi B.V., Amsterdam, The Netherlands), mounted on a headcap (extended international 10/20 layout, NeuroSpec Quick Cap) and referenced to the linked earlobes. The analysis has been focused on electrode sites POz, Pz, CPz, Cz, FCz, and Fz. This study is not aimed at performing source localization and we do not define regions of interest (ROIs) following the classical definition. Vertical and horizontal ocular movements were recorded using two pairs of bipolar electrodes placed beneath and above each eye next to the lateral canthi. The data acquisition (DC amplifiers and software by Biosemi, USA) was set with a sampling rate of 1,024 Hz at 24 bits resolution and band-passed filtered with a lower cutoff at 0.05 Hz and an upper cut-off at 200 Hz. Electrode impedances were checked and kept always below 20*kΩ* for all channels before starting the continuous recording of the EEG (Kappenman and Luck, 2010). The final checkup of the electrophysiological equipment and of the quality of brain signals was completed in about 30 min. The participants were instructed to maintain their gaze on a white fixation cross at the center of a 19-inch computer screen at a viewing distance of about 70 cm. At the begin of the recording session, the EEG was recorded during 2 min while the participants kept the eyes closed and during 2 min while they fixated a cross on the center of the computer screen.

The brain signals were preprocessed and analyzed with BrainVision Analyzer 2.0.4 (Brain Products, Gilching, Germany). Markers were used off-line to segment the continuous EEG data into epochs triggered by pressing the spacebar (the event *S*) and by clicking on the selected investment to gamble (the event *I*), as illustrated in Figure 1. For ERPs, the trials were cut into epochs lasting 1,500 ms ranging from −500 to +1,000 ms around the triggers (i.e., the events *S* and *I*). Visual inspection of the EEG was performed to remove immediately those epochs containing high amplitude muscle activity related noise, large eye blinks, and other easily identifiable artifacts. Infomax Independent Component Analysis was used to correct saccade-related eye movements whenever possible or to reject the epoch (Luck, 2014). The epochs were further scanned and inspected visually for contamination by residual minor artifacts. The total number of epochs recorded in the raw data was 2 triggers × 80 trials × 2 feedback frequency conditions, that is a total of 320 epochs per participant. The final number of EEG epochs preserved after the removal procedure was not different (unpaired Mann-Whitney Rank Sum Test *U* = 91, *p* = 0.11, *r* = 0.28) between controls (207, 211.4±12.4 epochs) and ADHD (239, 239.3±13.3 epochs). ERP analyses were performed on the artifact-free trials, band-pass filtered between 0.1 and 30 Hz (−12dB/octave). Subsequently, the trials were baseline corrected to the interval 500 ms prior to trigger onset and averaged for both conditions *LF* and *HF*. After removing all artifacts, the number of usable epochs for any particular ERP analysis had to be more than twenty.

## Results

### Personality traits and behavioral performance

HEXACO scores within each dimension of personality were determined for each subject and robust correlations were computed for each group (Table S1). The robust mixed-effects model with one within-subject factor (*personality*: H, E, X, A, C, O) and one between-subject factor (*groups*: controls and ADHD) revealed a significant interaction between the factors for openness [*t*_(34)_ = 5.96, *p* < 0.001, *r* = 0.71], conscientiousness [*t*_(34)_ = 3.89, *p* < 0.001, *r* = 0.55], extraversion [*t*_(34)_ = 3.27, *p* < 0.01, *r* = 0.49] and emotionality [*t*_(34)_ = 2.10, *p* < 0.05, *r* = 0.34]. In ADHD patients, we observed significant lower scores for conscientiousness, extraversion, and agreeableness (Table 1). In particular, agreeableness in the ADHD was correlated with higher risk indexes irrespective of the feedback frequency of the outcome [*t*_(16)_ = 2.61, *p* = 0.02, *d* = 0.10, Table S2].

**Figure 2:**
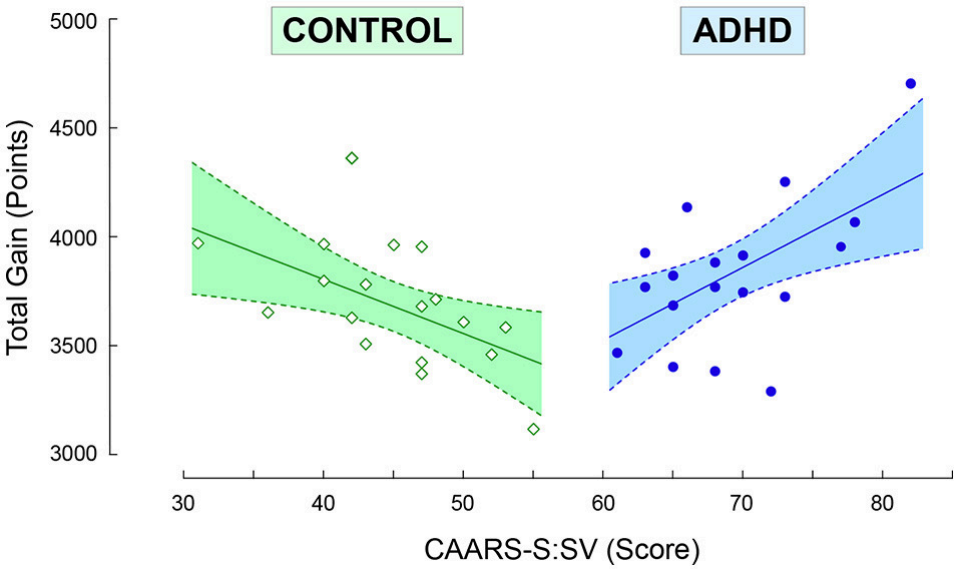
Scatter plot of the total gain *TotG*, cumulated during both feedback frequency conditions, as a function of CAARS, the normalized ADHD Index *T*-score. The robust regression equations for controls and ADHD are equal to *y* = 4, 800−24.7*x* [*F*_(1, 16)_ = 6.43, *p* = 0.02, *R*^2^ = 0.294] and *y* = 1541+33.2*x* [*F*_(1, 16)_ = 6.74, *p* = 0.02, *R*^2^ = 0.307]). All points were included for the robust regression. Each point represents the data from one participant. Dashed lines represents 95% confidence interval.

The overall amount of points *TotG* gained by the ADHD was not different from controls (Table 2). The robust statistics did not reveal any effect for gender (F_(1, 34)_ = 0.481, *p* = 0.49). However, a refined factorial analysis showed a very significant interaction between the group factor and the CAARS index on *TotG* [$\chi_{\left(4\right)}^{2}$ = 15.23, *p* < 0.001, *V* = 0.46]. The CAARS index was positively correlated in ADHD, and negatively correlated in controls, with the cumulated amount of points gained during both conditions (Figure 2). More specifically, in ADHD the risk index was positively correlated with CAARS-B, the hyperactive-impulsive symptoms subscale (Table S2). The participants of either group tended to keep the same risk-taking attitude in both feedback conditions (Figure S1). No gender effect was observed for the risk index [*F*_(1, 34)_ = 1.400, *p* = 0.24]). The response times of ADHD were slower than controls. In addition, the ADHD group was characterized by faster response times during *LF* than during *HF* trials (Table 2). No gender effect was observed for the reaction times neither during *LF* [*F*_(1, 34)_ = 0.823, *p* = 0.37] nor during *HF* trials [*F*_(1, 34)_ = 0.101, *p* = 0.75].

**Table 2:** Descriptive statistics (Median, mean, and SEM) of participants' behavioral performance during the probability gambling task.

	**Controls**	**ADHD**	**Between groups**
N	18	18	
**TOTAL GAIN (POINTS)**			
Within-condition			
• High frequency feedback	1,852	1,914	*U* = 187.5
*TG*(*HF*)	1,843 (43)	1,899 (60)	*p* = 0.43 *r* = 0.13
• Low frequency feedback	1,840	1,938	*U* = 197.5
*TG*(*LF*)	1,861 (43)	1,935 (46)	*p* = 0.27 *r* = 0.19
Within groups	*Z* = 0.07	*Z* = 0.74	
*Between conditions*	*p* = 0.96 *r* = 0.01	*p* = 0.48 *r* = 0.12	
**NORMALIZED RISK INDEX**			
Within-condition			
• High frequency feedback	−0.03	0.24	*U* = 186
*RI*(*HF*)	0.06 (0.10)	0.13 (0.10)	*p* = 0.46 *r* = 0.13
• Low frequency feedback	−0.06	0.25	*U* = 196.5
*RI*(*LF*)	−0.01 (0.12)	0.11 (0.12)	*p* = 0.28 *r* = 0.18
Within groups	*Z* = 1.18	*Z* = 0.26	
*Between conditions*	*p* = 0.25 *r* = 0.20	*p* = 0.81 *r* = 0.04	
**RESPONSE TIME (MS)**			
Within-condition			
• High frequency feedback	1,083	1,544	*U* = 204
*RT*(*HF*)	1,245 (146)	1,595 (196)	*p* = 0.19 *r* = 0.22
• Low frequency feedback	1,032	1,274	*U* = 197
*RT*(*LF*)	1,136 (153)	1,414 (200)	*p* = 0.28 *r* = 0.18
Within groups	*Z* = 1.13	*Z* = 2.33^*^	
*Between conditions*	*p* = 0.27 *r* = 0.19	*p* < 0.05 *r* = 0.39	

*“Between groups” comparison is assessed by the unpaired Mann-Whitney Rank Sum Test and the statistics U is reported. “Within group” (between conditions) comparison is assessed by the paired Wilcoxon signed rank test and the statistics Z is reported. Note that p-value equal to p-value and effect size r are reported for both tests*.

### Latencies of event related potentials components

**Figure 3:**
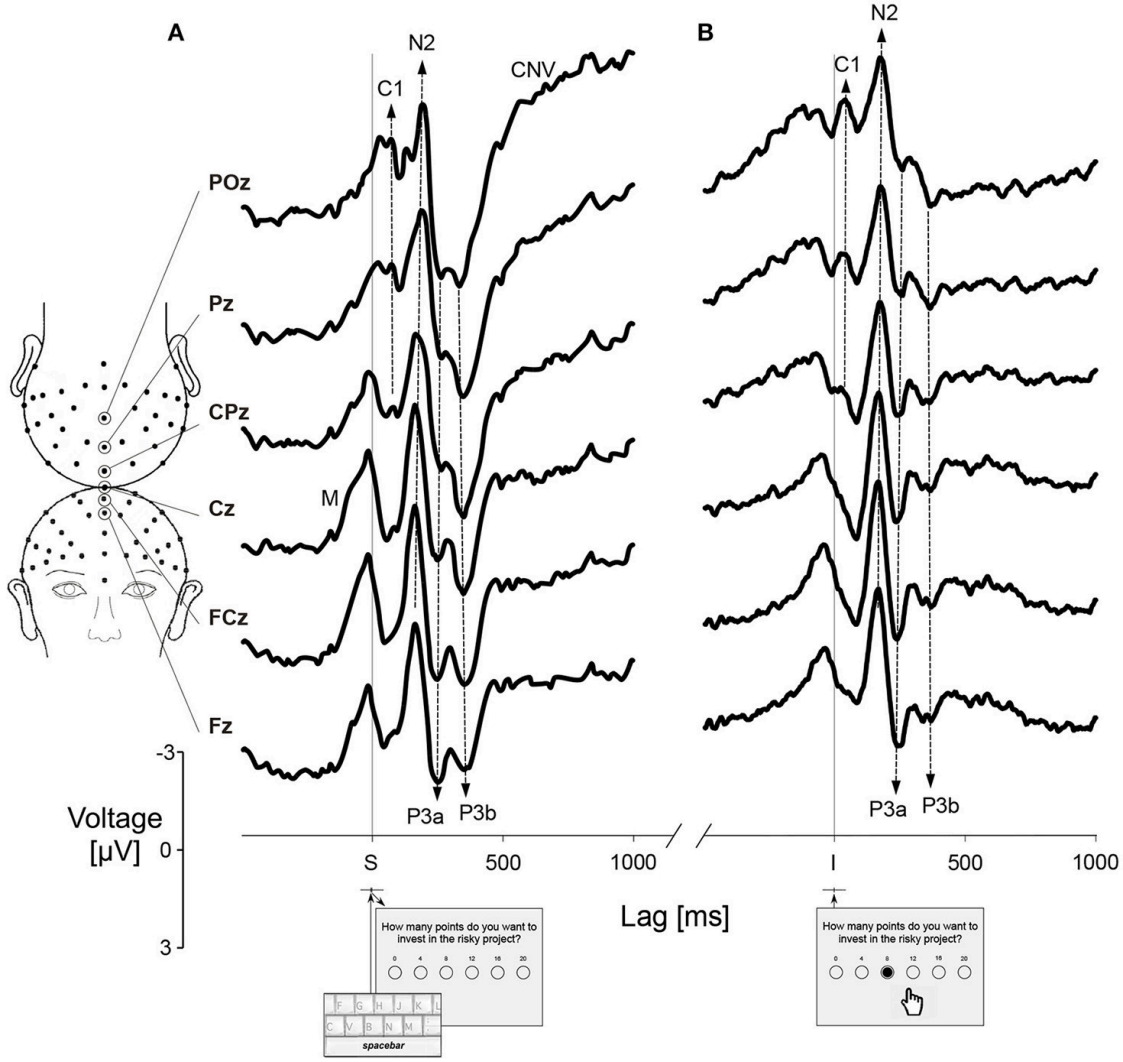
Event-Related Potentials (ERPs) during the probabilistic gambling task. Grand average at Fz, FCz, Cz, CPz, Pz, and POz sites for all participants (*N* = 32) and at all conditions (*HF, LF*) pooled together. **(A)** ERPs triggered by the event *S*, corresponding to the trial onset. **(B)** ERPs triggered by the event *I*, corresponding to the gambling choice. C1, visual evoked potential component; CNV, Contingent Negative Variation; M, premotor response; N2-P3a, the complex of components associated with expectation-attention-orienting processing; P3b, positive peak associated with the cognitive workload.

Four ADHD patients were discarded from the electrophysiological analyses because of excessive movements artifacts. The build-up of a premotor related brain activity (*M*) appeared about 150 ms before pressing the spacebar (the trigger event *S*) (Figure 3). An early visual event-related component C1, reflecting the initial response of the primary visual cortex, peaked near 70 ms after event S on POz and Pz. Both *S* and *I* triggering events evoked a negative-positive complex of peaks (N2-P3a) at approximately 175 and 250 ms. Immediately following P3a there was a second positive component (P3b), usually associated with a cognitive workload, at a latency near 340 ms. During the time interval between the events *S* and *I*, we observed a slow negative deflection after P3b, with greater amplitude toward the parietal areas. This component is usually referred to as CNV (contingent negative variation) and is not further analyzed in this study (Figure 3).

Simple effects of between-subject factor (*groups*: controls and ADHD) and within-subject factor (*conditions*: *HF* and *LF*) on the latencies of ERP components recorded at the frontocentral (Fz, FCz, and Cz pooled together) and centroparietal (CPz, Pz, and POz pooled together) sites were studied by robust statistics (Table 3). After the trial onset, P3a at frontocentral locations during the *LF* trials peaked earlier in the ADHD group than in the control group. In the ADHD, we observed a significant effect between low- and high-frequency feedback conditions at centroparietal sites for N2 and at frontocentral sites for P3b components (Table 3). These results show that the neural activity in the ADHD is affected by the frequency feedback condition of the protocol beginning with the trial onset (*S*) when the participant elaborates the gambling strategy. For main effects, we considered a linear mixed-effects model of the latency of an ERP component depending on two within-subject factors (*recording sites*: POz, Pz, CPz, Cz, FCz, and Fz; *conditions*: *HF* and *LF*) and one between-subject factor (*groups*: controls and ADHD). This model assumes that the latency of an ERP component is related to fixed effects, i.e., the interaction between the *groups, recording sites*, and *conditions* factors, and to additional independent random effects for each within-subject factor. In both groups, the analysis by the linear mixed-effects model revealed a significant effect of frequency feedback on P3a latency at frontocentral and centroparietal locations [$\chi_{\left(1\right)}^{2}$ = 10.75, *p* = 0.001, *V* = 0.17]. This effect might suggest that this ERP component is generated by a neural circuit associated with processing the frequency feedback outcome.

**Table 3:** Median and averaged ERPs latencies (*ms*) ± SEM at the frontocentral (Fz, FCz, CZ) and centroparietal (CPz, Pz, POz) sites during the high-frequency feedback (*HF*) and low-frequency feedback (*LF*) conditions after the trial onset and after the gambling choice.

		**Within-condition High-frequency feedback (HF)**			**Within-condition Low-frequency feedback (LF)**			**Within groups Between conditions (HF vs. LF)**	
		**Controls**	**ADHD**	**Between groups**	**Controls**	**ADHD**	**Between groups**	**Controls**	**ADHD**
**TRIAL ONSET**									
N2	Frontocentral	164.0	173.5	*U* = 1139	171.0	174.0	*U* = 1,180	*Z* = 1.22	*Z* = 0.84
		170.1 (2.9)	169.5 (2.3)	*p* = 0.97 *r* = 0.00	172.2 (2.1)	172.0 (2.5)	*p* = 0.74 *r* = 0.03	*p* = 0.23 *r* = 0.12	*p* = 0.41 *r* = 0.09
	Centroparietal	190.5	179.0	*U* = 905	189.5	187.0	*U* = 1,052	*Z* = 1.04	*Z* = 2.58^**^
		185.8 (3.4)	177.7 (3.4)	*p* = 0.09 *r* = 0.18	187.5 (2.8)	184.1 (3.4)	*p* = 0.55 *r* = 0.06	*p* = 0.30 *r* = 0.10	*p* < 0.01 *r* = 0.28
P3a	Frontocentral	246.5	243.0	*U* = 1116	262.0	247.0	*U* = 862.5^*^	*Z* = 3.48^***^	*Z* = 2.13^*^
		247.8 (3.3)	242.1 (3.9)	*p* = 0.90 *r* = 0.01	259.9 (3.7)	249.2 (3.2)	*p* < 0.05 *r* = 0.20	*p* < 0.001 *r* = 0.33	*p* < 0.05 *r* = 0.23
	Centroparietal	251.0	255.5	*U* = 1068.5	264.0	260.0	*U* = 997	*Z* = 3.31^***^	*Z* = 2.85^**^
		258.3 (3.4)	248.9 (5.2)	*p* = 0.63 *r* = 0.05	266.0 (3.2)	260.0 (3.4)	*p* = 0.31 *r* = 0.10	*p* < 0.001 *r* = 0.32	*p* < 0.01 *r* = 0.31
P3b	Frontocentral	351.0	349.0	*U* = 974	356.0	363.5	*U* = 1,121	*Z* = 1.78	*Z* = 2.90^**^
		352.6 (3.7)	346.7 (3.4)	*p* = 0.46 *r* = 0.08	358.2 (2.7)	354.3 (4.3)	*p* = 0.95 *r* = 0.01	*p* = 0.08 *r* = 0.17	*p* < 0.01 *r* = 0.32
	Centroparietal	353.0	348.0	*U* = 951.5	357.0	354.0	*U* = 1,054	*Z* = 0.64	*Z* = 1.86
		356.9 (3.8)	348.0 (3.7)	*p* = 0.18 *r* = 0.14	360.4 (3.4)	353.3 (4.6)	*p* = 0.56 *r* = 0.01	*p* = 0.53 *r* = 0.06	*p* = 0.06 *r* = 0.20
**GAMBLING CHOICE**									
N2	Frontocentral	167.5	174.0	*U* = 1,414.5^*^	165.0	174.5	*U* = 1,542.5^**^	*Z* = 1.40	*Z* = 0.81
		168.1 (2.2)	176.2 (2.4)	*p* < 0.05 *r* = 0.21	167.5 (1.8)	176.0 (2.0)	*p* < 0.01 *r* = 0.31	*p* = 0.16 *r* = 0.13	*p* = 0.43 *r* = 0.09
	Centroparietal	178.5	181.0	*U* = 1,298.5	178.0	184.0	*U* = 1,425.5^*^	*Z* = 1.08	*Z* = 60
		177.7 (2.5)	183.3 (2.6)	*p* = 0.23 *r* = 0.12	175.8 (2.6)	184.1 (2.6)	*p* < 0.05 *r* = 0.22	*p* = 0.28 *r* = 0.10	*p* = 0.55 *r* = 0.07
P3a	Frontocentral	235.0	242.0	*U* = 1,366	237.5	245.0	*U* = 1,228	*Z* = 1.37	*Z* = 0.51
		239.3 (2.0)	245.1 (2.8)	*p* = 0.09 *r* = 0.18	241.8 (2.0)	243.6 (2.7)	*p* = 0.49 *r* = 0.07	*p* = 0.17 *r* = 0.13	*p* = 0.61 *r* = 0.06
	Centroparietal	244.0	245.0	*U* = 1,223.5	239.5	251.5	*U* = 1,354.5	*Z* = 0.55	*Z* = 0.53
		244.1 (2.4)	249.5 (3.3)	*p* = 0.51 *r* = 0.07	242.7 (2.5)	247.7 (4.0)	*p* = 0.10 *r* = 0.17	*p* = 0.59 *r* = 0.05	*p* = 0.61 *r* = 0.06
P3b	Frontocentral	351.0	342.0	*U* = 815.5^*^	360.0	350.5	*U* = 892.5	*Z* = 1.42	*Z* = 1.28
		354.9 (3.2)	347.7 (3.1)	*p* < 0.05 *r* = 0.23	360.5 (3.3)	353.1 (3.6)	*p* = 0.07 *r* = 0.18	*p* = 0.16 *r* = 0.14	*p* = 0.20 *r* = 0.14
	Centroparietal	360.0	350.5	*U* = 768^**^	370.5	362.0	*U* = 1,100	*Z* = 0.52	*Z* = 2.29^*^
		365.1 (2.9)	353.8 (3.1)	*p* < 0.01 *r* = 0.28	364.6 (3.2)	363.5 (4.2)	*p* = 0.80 *r* = 0.03	*p* = 0.61 *r* = 0.05	*p* < 0.05 *r* = 0.25

*“Between groups” comparison is assessed by the unpaired Mann-Whitney Rank Sum Test and the statistics U is reported. “Within group” (between conditions) comparison is assessed by the paired Wilcoxon signed rank test and the statistics Z is reported. Two-sided p-value and effect size r are reported for both tests*.

After the gambling choice, the linear mixed-effects model revealed a significant main effect of the electrode factor for N2 [$\chi_{\left(5\right)}^{2}$ = 46.58, *p* < 0.001, *V* = 0.16]. The N2 component, after pooling all electrodes and both frequency feedback conditions, peaked later in the ADHD than controls (*U* = 22,557, *p* < 0.001, *r* = 0.21). The peak of P3a tended also to occur later in the ADHD than controls (*U* = 20,624, *p* < 0.05, *r* = 0.12). On the opposite, notice that P3b peaked earlier in ADHD than controls (after pooling all electrodes and both conditions: *U* = 1,4374, *p* < 0.001, *r* = 0.18), in particular during the *HF* condition (Table 3).

### Differential waveform analysis

**Figure 4:**
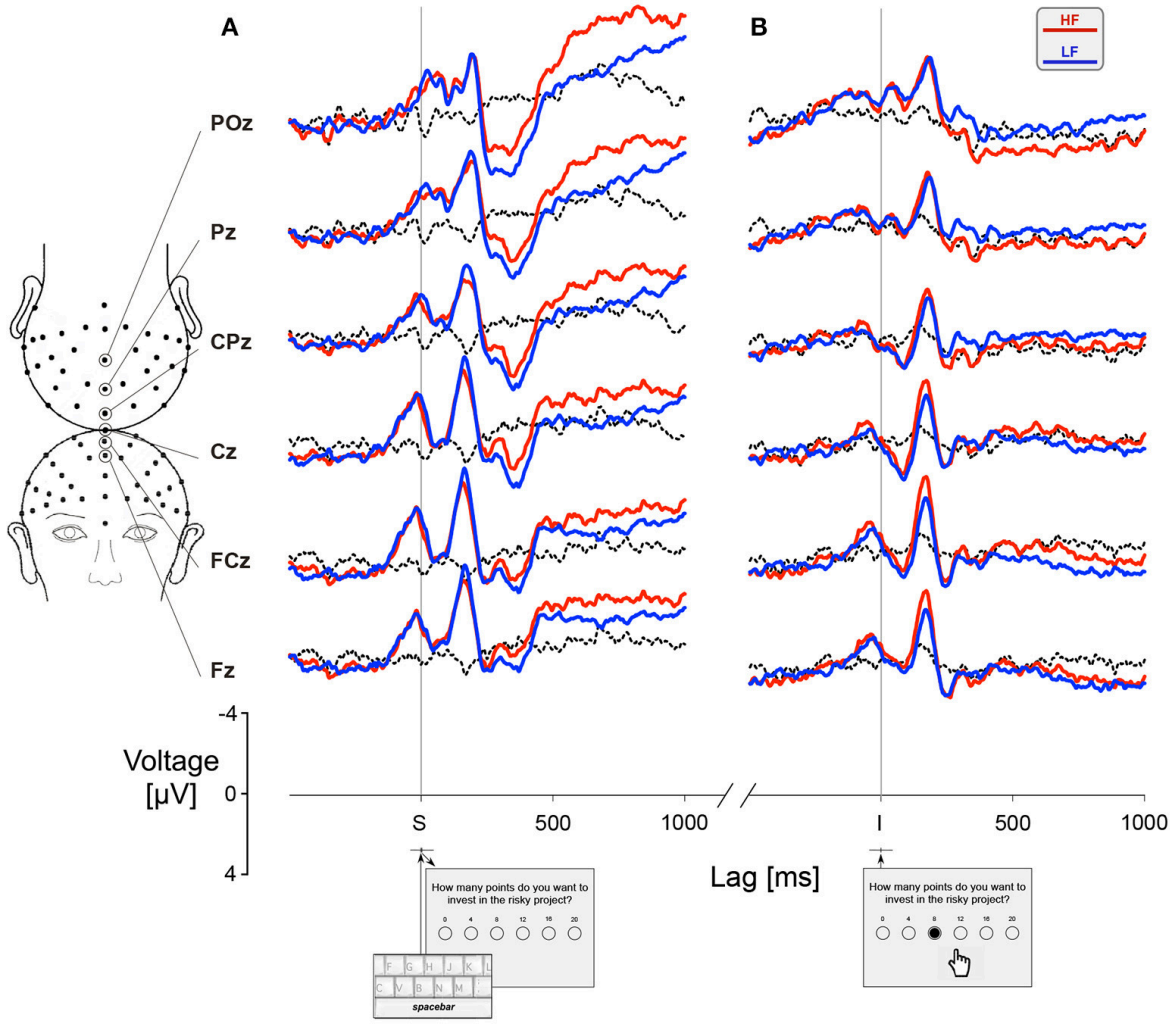
Effect of feedback condition on the grand-averaged ERPs. **(A)** ERPs averaged for all participants (*N* = 32) at electrodes sites POz, Pz, CPz, Cz, FCz, and Fz across conditions *HF* (red lines) and *LF* (blue lines) after the trial onset (*S*). **(B)** ERPs after the gambling choice (*I*). Dotted lines correspond to the difference waves, computed by subtracting *LF* curves from *HF* curves.

**Figure 5:**
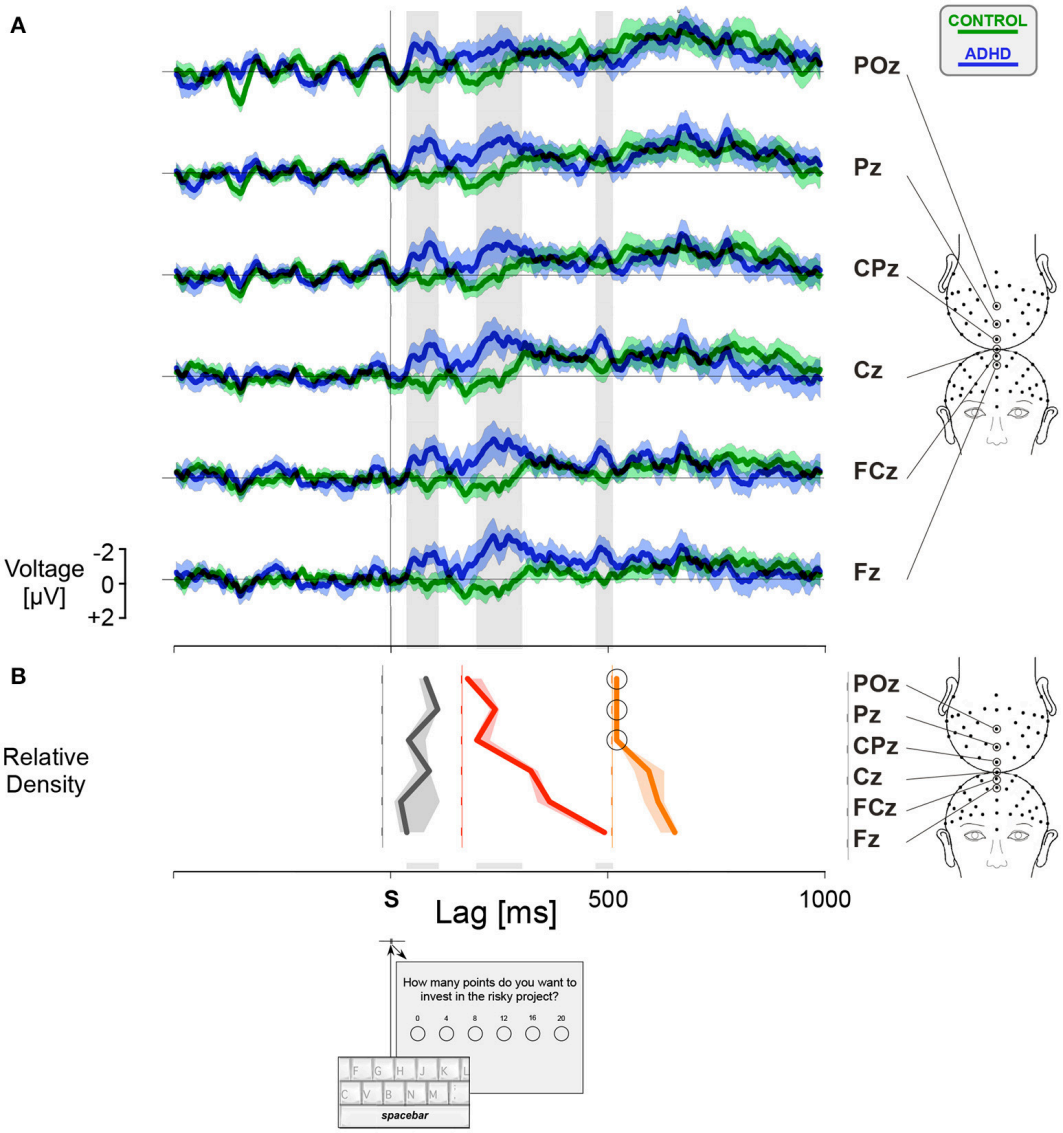
Feedback related differential activities triggered by the trial onset (*S*). **(A)** The curves were computed by subtracting ERPs associated with *LF* from ERPs associated with *HF* for controls (green lines, *N* = 18) and ADHD participants (blue lines, *N* = 14). The confidence interval (mean curve ± SEM) is shown by the shaded areas. **(B)** The relative density plot shows the spatial distribution of the estimated amplitude of the differential curves integrated along three intervals emphasized by the gray stripes, centered at latencies 80 ms (C1, dark gray), 260 ms (N2-P3, red), and 490 ms (N500, orange). The shaded areas correspond to non-parametric estimation of the 95% confidence intervals. Circles show those values not significantly different from zero. See Table S3 for more details.

**Figure 6:**
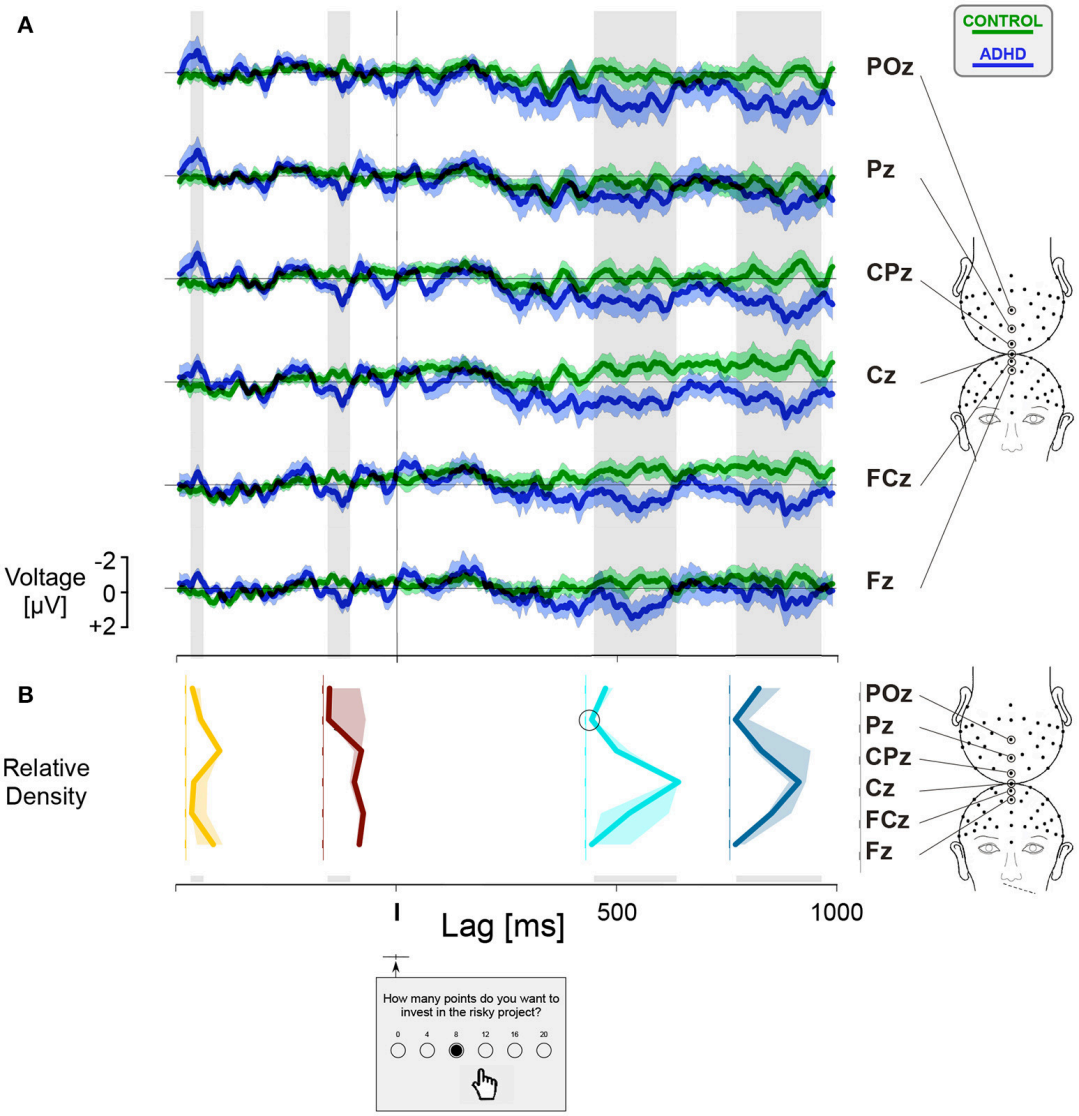
Feedback related differential activities triggered by the gambling choice (*I*). **(A)** The curves were computed in the same way as Figure 5A. **(B)** The relative density plot shows the spatial distribution of the estimated amplitude of the differential curves integrated along four intervals emphasized by the gray stripes, centered at latencies 450 ms (yellow) and 140 ms (brown) before the gambling choice and 490 ms (N400-like, turquoise) and 850 ms (LPP, teal blue) after the gambling choice. Circles show those values not significantly different from zero. The shaded areas correspond to non-parametric estimation of the 95% confidence intervals. See Table S3 for more details.

For each participant, we calculated separately the ERPs for *HF* and *LF* trials. The feedback related differential ERPs were obtained by subtracting the ERP recorded during *LF* trials from the ERP recorded during *HF* trials, as illustrated by the dotted lines in Figure 4. In order to assess the group factor, we compared the feedback related differential ERPs for controls and ADHD participants triggered by the trial onset (Figure 5) and by the gambling choice (Figure 6) at six electrode sites. Confidence intervals were computed around the grand average feedback related differential ERPs curves for each group, represented by green shaded areas for controls and blue shaded areas for ADHD patients. The intersection between the two shaded areas allows to estimate whether a group factor is associated with feedback related differential brain activities. A complete overlap between the shaded areas is a sign of no difference between controls and ADHD. On the opposite, the integration of an area delimited by limit lines of the corresponding confidence areas over selected time intervals allows to estimate the existence of a group factor. Non-parametric statistical analyses were performed using Wilcoxon signed rank tests (see Table S3 for details).

After the trial onset, the differences between groups appeared for an interval centered at a latency of 80 ms, corresponding to the C1 wave, and for an interval centered at latency 260 ms, corresponding to the N2-P3 complex (Figure 5A, Table S3). For such N2-P3 component, we observed that controls were characterized by a greater amplitude of the N2 wave in *LF* compared to *HF*, whereas the opposite effect occurred in ADHD participants. Notice that the difference between ADHD and controls became increasingly significant toward the frontal locations, peaking at site Fz (Figure 5B, red relative density curve, Table S3). After trial onset, an N500 component, also related to the feedback frequency condition, determined a significant differential interval peaking toward the frontal locations at a latency near 500 ms (Figure 5B, orange curve, Table S3). This is the kind of ERP component expected to occur in decision-making tasks characterized by risky decisions vs. conservative responses.

Further significant “between groups” difference in feedback frequency related activities appeared centered at 450 ms before making the gambling choice (trigger *I*) along the frontocentral sites (Figure 6B, yellow curve, Table S3). In ADHD this component was characterized by a greater amplitude of ERP during *HF* trials, likely to be associated with the motivation and selection of a risky goal-directed behavior. The differences “between groups” in movement initiation, toward the button associated with the selected gamble, was revealed in the ADHD by larger feedback frequency related activity at the parietal site Pz ~ 140 ms before the gambling choice (Figure 6B, brown curve, Table S3). The differential waveform analysis showed differences between ADHD and controls for two intervals characterized by a sharp frontocentral scalp distribution and by greater amplitudes during the *LF* condition in the ADHD group (Figure 6B, cyan, and blue curves). An ERP component, corresponding to the N400-like wave, peaked at 490 ms after the event *I* and lasting more than 200 ms (Table S3). This wave is strongly associated with the postdecisional evaluation of the choice made when gambling. The last component corresponded to an LPP lasting between approximately 770 and 950 ms after the gambling selection (Table S3). The LPP is a characteristic marker of emotion regulation and affective stake of the decision-making processes. In particular, notice at sites CPz, Cz, and FCz the opposite trend of the feedback frequency differential ERP curves (Figure 6A) for ADHD (blue lines, mainly toward positive values) and controls (green lines, mainly toward negative values).

## Discussion

To the best of our knowledge, this is the first study aimed at investigating the behavior and the ERPs elicited by a PGT in young ADHD adults without clinically assessed comorbid disorders in the context of personality assessed by the HEXACO-Personality Inventory. In both ADHD and controls, the risks taking strategies varied greatly between individuals and were not affected by the feedback frequency of the gambling outcome. On the contrary, characteristic ERP components triggered by the trial onset and by gamble selection appear as valuable markers to distinguish ADHD from controls. The manipulation of outcomes' feedback frequency showed that at trial onset the N2-P3a components, associated with expectation-association-orienting processing, peaked earlier in the ADHD than in controls. The subsequent build-up of the response by ADHD patients was characterized by an increase in response time till gamble selection and, after the choice was made, the N2-P3a complex occurred later, especially toward frontal sites. Such increases in latencies could be associated with competing for mutual inhibitory networks that delay execution-related processes. After gambling, we observed that the P3b component peaked earlier in the ADHD than in controls followed by a distinct pattern of N400-like and LPP components. In the young ADHD adults, we raise the hypothesis that the activity of frontocentral and centroparietal neural circuits drive the decision-making processes dictated by an impaired cognitive workload followed by the build-up of large emotional feelings generated by the conflict toward the outcome of the gambling choice.

### Gender effect

Gender differences in functional maturation of brain networks involved in early stages of sensorimotor processing have been reported in preadolescent children (Nanova et al., 2011). Such gender effect might have an important impact on ADHD studies because of electrophysiological evidence for abnormal preparatory states and inhibitory processing in children (Yordanova et al., 2001) and adults affected by ADHD (McLoughlin et al., 2010). Higher rates of self-reported anxiety symptoms in females with ADHD were observed in one study (Skogli et al., 2013). Hence, due to the limited size of our sample, we searched for a potential bias associated with an effect of gender. No significant main gender effect was observed neither for self-reported scales (CAARS and ASRS) nor for the risk taking and gambling behavior, in agreement with functional (Disney et al., 1999; Rucklidge, 2008; Simon et al., 2009) and anatomical studies of young ADHD adults (Yang et al., 2008). The absence of a gender effect in young adults emphasizes the subsequent group effects observed in the analyses of this study.

### Personality

We observed lower conscientiousness, agreeableness and extraversion scores in ADHD than in controls, generally confirming previous studies based on the “Big Five” model (BF). Those studies reported ADHD patients characterized by low scores of conscientiousness and agreeableness and a high score of neuroticism (Nigg et al., 2002; Parker et al., 2004; Jacob et al., 2007; Miller et al., 2008). Several studies performed in groups other than ADHD patients have highlighted an association between low agreeableness and gambling behavior or risk taking (de Vries et al., 2009; Fang and Mowen, 2009; Tackett et al., 2015). Notice that conscientiousness and extraversion are defined in the same way in both BF and HEXACO models, but agreeableness is only partially overlapping. Low conscientiousness was reported being strongly related with inattention and disorganization (Nigg et al., 2002; Parker et al., 2004) and low extraversion with ADHD inattentive subjects (Parker et al., 2004; Jacob et al., 2007). In our study, the CAARS index of ADHD was correlated with extraversion (Table S1). These results are coherent with the fact that our ADHD group was strongly characterized by inattentive symptoms, with an average *T*-score of 79.6 (Table 1), a high score comparable only with the ADHD group reported by another study (Fisher et al., 2011). A one-tail test showed that the score to openness-to-experience in ADHD was higher than in controls, while most of other studies did not report any significant result regarding openness, whose definition overlaps in HEXACO and BF models. It is noticeable that the association of high scores of openness with risk taking and sensation seeking behaviors was reported elsewhere also in participants not affected by ADHD (de Vries et al., 2009).

### Risk taking and gambling behavior

The Iowa Gambling Task, the Balloon Analog Risk Task, and the Game of Dice Task could not reveal any salient group effect between ADHD adults and controls (Ibanez et al., 2012; Groen et al., 2013) and is in agreement with behavioral data reported from ADHD children and adolescents (Yordanova et al., 2011). The probabilistic gambling task used in the current study is an explicit task with two conditions of feedback frequency of the outcome. Overall, and in each condition separately, we could not find any significant difference “between groups” neither in total gains nor in the risk index. However, we observed an interaction of CAARS score with the total earning: the lower the score the lower the gains in ADHD participants, but the lower the score the higher the gains in controls.

ADHD patients are characterized by an increased likelihood to take greater risks than age-matched controls in activities such as extreme driving and substance abuse (Barkley et al., 1996; Barkley and Fischer, 2010; Lee et al., 2011). It is recognized that childhood ADHD history has a strong influence on persistent pathological gambling (Breyer et al., 2009). Recent findings point out that pathological gambling in adulthood is associated with a comparably elevated level of impulsiveness in ADHD and non-ADHD gamblers (Dai et al., 2016). Our ADHD group was mainly characterized by inattentiveness rather than impulsiveness, which may explain why the “between groups” comparison could not immediately reveal a significant difference in risk-taking behavior between ADHD and controls. Subtype of ADHD affects the pattern of performance differences between controls and ADHD patients (O'Driscoll et al., 2005; Derefinko et al., 2008; Gorman Bozorgpour et al., 2013), in particular with an association between the ADHD/inattentive subtype with slower response times but similar response accuracy with respect to controls.

We observed that both risk strategy and the total gains were not significantly affected by feedback frequency in either group of participants. However, the investment of higher stakes associated with a low-frequency presentation of the outcomes was observed in the original Gneezy and Potters' (Gneezy and Potters, 1997), and other tasks (Bellemare et al., 2005; Langer and Weber, 2008). In the original task (Gneezy and Potters, 1997), the participants had to choose one bet per block in the *LF* condition, whereas the participants of the current study were allowed to gamble independently at each trial. Hence, differences in experimental design and protocol may also contribute to explain our result.

### Neural dynamics and event related potentials

The manipulation of the feedback frequency of the outcome necessarily affects how individuals tend to evaluate each transaction in combination and not separately to the previous ones. The recent introduction of neurofeedback training in children with ADHD (Heinrich et al., 2014; Marx et al., 2014; Zuberer et al., 2015) raises the question whether young adults affected by ADHD show differences in ERPs associated with feedback frequency of the outcome. The PGT is characterized by a free-operant (self-paced) behavior given that the trial onset is associated with pressing the keyboard spacebar. In this goal-directed task, the participants are informed that they play trials alternatively distributed in blocks of low and high feedback frequency of the outcome. Hence, it is likely that the participants develop their most adapted cognitive strategy at trial onset, by balancing the costs and benefits of making a decision regarding the amount to gamble (Hilbig and Pohl, 2009; Glöckner et al., 2014). The interval between the trial onset and the time of choosing the amount to gamble may be interpreted as a cue-target interval, given that the selected gamble is a target of a self-paced movement. In the ADHD group exclusively, we observed a feedback frequency effect on response times, i.e., faster response times during *LF* than during *HF* trials. These differences might be due to inhibitory control deficits in ADHD (McLoughlin et al., 2010; Ibanez et al., 2012; Cross-Villasana et al., 2015; Roberts et al., 2016) and the decision-making processes during PGT are likely to be associated with distinct brain network dynamics (De La Fuente et al., 2013).

Differences in P3 waveforms associated with levels of hyperactivity-impulsivity have been reported in the literature (Johnstone et al., 2003; Alexander et al., 2008). During *HF* trials, the P3a component peaked earlier in both groups at all sites. Our ADHD group was mainly characterized by inattentiveness, thus suggesting that it is the rationale to expect smaller differences in P3a during *HF* trials and larger differences during *LF* trials. The P3a component is associated with stimulus-driven attention engagement (Polich, 2007) and its latency is likely to increase with an increased demand of an active orienting process associated with a low feedback frequency of the outcome. Frontal areas and the insula contribute mainly to P3a (Bledowski et al., 2004) and during *LF* trials we found differences between groups restricted to the frontocentral sites. Other studies in ADHD have also reported attenuated P3a (Yang et al., 2015) and earlier P3a latencies (Rodriguez and Baylis, 2007). Hence, we may infer that reduced latencies for P3a wave associated with stimulus-driven attention may be associated with activation of a brain network less extended in ADHD than in controls. In our ADHD group, we observed that P3b peaked earlier and a N500 wave, with a large amplitude centered on 490 ms, characterized the ERP at frontocentral sites during *HF* trials. The topographical distribution and latency of our N500 component are in agreement with the wave related to emotional tension-resolution patterns and response selection described elsewhere in risky decision making in gambling tasks (Yang et al., 2007; Mennes et al., 2008; Steinbeis and Koelsch, 2008; Chen et al., 2010). Later in the cue-target interval, during movement initiation toward reaching the target (i.e., clicking on the selected amount to gamble), the ADHD were characterized during *LF* trials by a frontocentral wave at −450 ms and a centroparietal wave at −140 ms. In the ADHD, the neural dynamics responsible for earlier response times during *LF* trials might be associated with the generation of these ERP components, in particular with the frontostriatal network supporting inhibitory control (Aron et al., 2004; Mennes et al., 2011; Sonuga-Barke and Fairchild, 2012) and the centroparietal network involved in processing memory related emotional cues (Weymar et al., 2011; Cona et al., 2015). Impairment of memory functions in ADHD is further supported by the finding of a smaller volume of the hippocampus reported for this kind of patients (Onnink et al., 2014). In our study, *LF* trials require more information to be retained in order to make inferences about the gambling. Then, altered memory capacity in ADHD is likely to affect their ability to process the information available during *LF* trials and to increase the emotional level of the response conflict.

Following the gamble selection, the N2-P3a component peaked later in ADHD compared to controls, in particular at frontocentral locations. This is in agreement with the enhancement of negative peak waveforms (N2 or FRN, Feedback-Related Negativity) reported in adult ADHD patients (Thoma et al., 2015) performing a probabilistic monetary task bearing some similarities with our PGT. If we assume that this ERP complex is associated with the brain network engaged in the stimulus-driven attention it is likely that the emotional representation of the gambling choice in the ADHD (Yang et al., 2007; Mennes et al., 2008) may involve an extension of the processing network, thus resulting in larger N2-P3a latencies. After P3b, a negative deflection centered on 490 ms with larger amplitude during *LF* than *HF* trials was characteristic of ADHD. This feedback frequency related activity peaked over Cz and is likely to be associated with an N400-like ERP component related to a contextual mismatch (Kutas and Federmeier, 2011). In the present task, this internal event is represented by the conceptual processing of the gambling outcome expectation, in agreement with other observations of N400-like waves peaking over central areas (Polezzi et al., 2010; Yang and Zhang, 2011). The last feedback frequency related event that distinguished ADHD and controls occurred at centroparietal sites at latencies in the range 760–930 ms after gambling selection. This component could be identified with the late positive potential (LPP) detected in various experimental designs related to the processing of affective content (Ferrari et al., 2008; Mesrobian et al., 2015) The finding of consistent group differences over central and centroparietal sites at different delays of the task suggest that deficits in young ADHD adults were not restricted to the inhibitory processes of the task, but were associated also with execution-related processes, as suggested by previous findings in ADHD children and adolescents (Gow et al., 2012; Singhal et al., 2012; Bunford et al., 2017). In ADHD participants, the low feedback frequency of the outcome produced an emotional reaction and a greater conflict toward the outcome of the choice. The time course of N400-like and LPP might be associated with the difficulty for the participants to know the accuracy of their choice until a feedback stimulus occurred at the end of the trial. This difference with controls appears in agreement with the characteristic post-error behavior reported in ADHD adolescents (Yordanova et al., 2011). By the time feedback occurred, any response conflict had dissipated, which suggests considering these two ERP components being associated with the postdecisional evaluation of the choice made.

### Conclusion

Event Related Potentials elicited by a PGT are valuable markers of decision-making deficits related to ADHD, more sensitive than classical behavioral markers based on the total gain, gambling amount, and risk-taking indexes. We used two conditions characterized by a low and high frequency of feedback information of the outcome. The manipulation of outcomes' feedback frequency showed that at trial onset the N2-P3a components, associated with expectation-association-orienting processing, peaked earlier in ADHD than in controls. Our results extend previous evidence in ADHD children and adolescents that deficits in the executive attention network, responsible for error processing and conflict monitoring, and the dissociation between perceptual and response conflicts, selectively modify the neural dynamics involved in decision-making processes.

## Author contributions

Authors SM, AL, and AV contributed equally to all stages of this work. MB contributed the psychiatric assessment and screening of the participants. LG contributed to the experimental design of the behavioral task.

### Conflict of interest statement

The authors declare that the research was conducted in the absence of any commercial or financial relationships that could be construed as a potential conflict of interest.
