# Supplementary material for: Event-Related Potentials during a Gambling Task in Young Adults with Attention-Deficit/Hyperactivity Disorder
Source: Front Hum Neurosci. 2018 Feb 27;12:79. doi: 10.3389/fnhum.2018.00079 (PMC5835343; doi:10.3389/fnhum.2018.00079)
Supplement: Supplementary file 1 [file Table1.pdf]

Table S1. Robust correlations among personality traits and ADHD assessment scales.

	ADHD ($N = 18$)									
Controls ($N = 18$)	H	E	X	A	C	O	CAARS	CAARS-A	CAARS-B	ASRS
H	.	.270	−.012	.512*	.263	−.160	−.085	.047	.211	.148
E	.124	.	−.052	.108	.344	.296	.479*	.292	−.097	.088
X	.024	−.321	.	−.146	−.047	.363	−.079	−.193	.128	.082
A	.333	.257	.057	.	.024	.170	−.278	−.200	−.245	−.132
C	.514*	.146	.120	.073	.	−.267	.018	−.023	.096	.264
O	.215	−.070	.400	.271	−.249	.	.256	−.021	−.325	−.031
CAARS	−.136	.291	−.327	.035	−.029	−.478*	.	.195	.052	.464*
CAARS-A	−.102	.123	−.151	.390	−.426	−.023	.587*	.	.391	.396
CAARS-B	−.432	.085	−.328	−.009	−.194	−.560*	.786**	.550*	.	.431
ASRS	−.301	.218	−.197	.048	−.209	−.299	.513*	−.373	.570*	.

Personality traits: [H]onesty-Humility, [E]motionality, e[X]traversion, [A]greeableness, [C]onscientiousness, [O]penness to experience). CAARS: ADHD Index; CAARS-A: DSM IV Inattentive Symptoms Subscale; CAARS-B: DSM IV Hyperactive-Impulsive Symptoms Subscale.

Robust correlation coefficients $\hat{\rho}_G$ following the Gaussian rank correlation estimators (Boudt et al., 2012).

(*) level of significance of $p<.05$; (**) level of significance of $p<.01$.
