# Supplementary material for: Event-Related Potentials during a Gambling Task in Young Adults with Attention-Deficit/Hyperactivity Disorder
Source: Front Hum Neurosci. 2018 Feb 27;12:79. doi: 10.3389/fnhum.2018.00079 (PMC5835343; doi:10.3389/fnhum.2018.00079)
Supplement: Supplementary file 2 [file Table2.pdf]

Mesrobian SK, Villa AEP, Bader M, Götte L and Lintas A (2018) Event-Related Potentials during a Gambling Task in Young Adults with Attention-Deficit/Hyperactivity Disorder. Front. Hum. Neurosci. 12:79. doi: 10.3389/fnhum.2018.00079

Table S2. Robust correlations between personality traits with measures of performance to PGT.

		H	E	X	A	C	O	CAARS	CAARS-A	CAARS-B	ASRS
Controls ($N = 18$)	$TotG$	.168	−.188	−.057	.197	.157	.502*	−.655**	−.383	−.648**	−.368
	TG(*HF*)	.012	−.067	−.229	.117	−.132	.519*	−.504*	−.133	−.442	−.317
	TG(*LF*)	.065	−.274	.070	.146	.218	.236	−.391	−.293	−.410	−.244
	RI	.139	−.332	.041	.055	.197	.245	−.280	−.045	−.196	−.161
	RI(*HF*)	.168	−.289	.119	.158	.124	.339	−.232	−.089	−.155	−.075
	RI(*LF*)	−.004	−.373	−.009	−.113	.215	.049	−.231	−.231	−.130	−.098
	RT	.435	.395	−.070	.011	−.062	.103	.054	.126	−.124	.319
	RT(*HF*)	.462*	.335	.021	−.058	−.031	.157	.001	.039	−.180	.222
	RT(*LF*)	.584*	.214	−.163	−.007	−.082	.010	−.011	−.091	−.263	.178
ADHD ($N = 18$)	$TotG$	.143	−.062	.021	.153	−.303	.113	.433	.014	−.062	.192
	TG(*HF*)	.073	−.179	.083	−.008	−.020	−.074	.227	.256	−.095	.419
	TG(*LF*)	.109	.173	.009	.044	−.414	.247	.530*	−.228	−.049	−.060
	RI	−.263	−.257	.312	−.567*	.072	−.130	.294	.090	.498*	.244
	RI(*HF*)	−.259	−.293	.394	−.563*	.081	−.136	.290	.070	.534*	.364
	RI(*LF*)	−.221	−.215	.281	−.542*	.011	−.080	.295	.110	.459	.139
	RT	.030	−.297	.004	−.099	.179	−.206	−.290	−.104	−.093	−.407
	RT(*HF*)	.085	−.266	.068	−.044	.171	−.221	−.283	−.116	−.040	−.441
	RT(*LF*)	.151	−.337	−.026	−.078	.232	−.326	−.310	−.156	−.115	−.319

Robust correlation coefficients $\hat{\rho}_G$ following the Gaussian rank correlation estimators (Boudt et al., 2012).
(*) level of significance of *p*<.05 ; (**) level of significance of *p*<.01.
