# Supplementary material for: Event-Related Potentials during a Gambling Task in Young Adults with Attention-Deficit/Hyperactivity Disorder
Source: Front Hum Neurosci. 2018 Feb 27;12:79. doi: 10.3389/fnhum.2018.00079 (PMC5835343; doi:10.3389/fnhum.2018.00079)
Supplement: Supplementary file 3 [file Table3.pdf]

Mesrobian SK, Villa AEP, Bader M, Götte L and Lintas A (2018) Event-Related Potentials during a Gambling Task in Young Adults with Attention-Deficit/Hyperactivity Disorder. Front. Hum. Neurosci. 12:79. doi: 10.3389/fnhum.2018.00079

Table S3. Differential waveform analysis for selected time intervals. Control and ADHD groups were analyzed during the high-frequency and low-frequency feedback conditions after the trial onset and after the gambling choice. Then, the density of the 'between groups' difference of the grand average ERPs was estimated using by the Hodges-Lehmann 2-sample estimator.

Trigger trial onset (S)	Interval (46:107 ms)		Interval (197:298 ms)		Interval (470:508 ms)	
	HL2 Density	*Between groups*	HL2 Density	*Between groups*	HL2 Density	*Between groups*
POz	.320	$Z = 6.82$ $p < .001$ $r = .60$	.000	$Z = 4.78$ $p < .001$ $r = .33$	.000	$Z =$ N/A N/A
Pz	.421	$Z = 6.94$ $p < .001$ $r = .61$	.236	$Z = 8.35$ $p < .001$ $r = .58$	.000	$Z =$ N/A N/A
CPz	.173	$Z = 6.60$ $p < .001$ $r = .58$	.079	$Z = 7.75$ $p < .001$ $r = .54$	.000	$Z = 2.00$ $p = .05$ $r = .22$
Cz	.350	$Z = 6.96$ $p < .001$ $r = .62$	.540	$Z = 8.78$ $p < .001$ $r = .61$	.274	$Z = 4.92$ $p < .001$ $r = .55$
FCz	.105	$Z = 6.12$ $p < .001$ $r = .54$	.699	$Z = 8.78$ $p < .001$ $r = .61$	.353	$Z = 5.19$ $p < .001$ $r = .58$
Fz	.158	$Z = 6.56$ $p < .001$ $r = .58$	1.167	$Z = 8.84$ $p < .001$ $r = .61$	.496	$Z = 5.24$ $p < .001$ $r = .59$

Trigger gambling choice (I)	Interval (-467:-440 ms)		Interval (-157:-108 ms)		Interval (446:630 ms)		Interval (767:958 ms)	
	HL2 Density	*Between groups*	HL2 Density	*Between groups*	HL2 Density	*Between groups*	HL2 Density	*Between groups*
POz	.006	$Z = 3.68$ $p < .001$ $r = .49$	.008	$Z = 5.00$ $p < .001$ $r = .50$	.116	$Z = 10.89$ $p < .001$ $r = .56$	.197	$Z = 11.00$ $p < .001$ $r = .55$
Pz	.077	$Z = 4.27$ $p < .001$ $r = .57$	.000	$Z = 4.73$ $p < .001$ $r = .47$	.000	$Z = 1.41$ $p = .19$ $r = .07$	.000	$Z = 3.74$ $p < .01$ $r = .19$
CPz	.236	$Z = 4.62$ $p < .001$ $r = .62$	.276	$Z = 6.20$ $p < .001$ $r = .61$	.208	$Z = 10.82$ $p < .001$ $r = .56$	.219	$Z = 10.73$ $p < .001$ $r = .54$
Cz	.018	$Z = 4.10$ $p < .001$ $r = .55$	.216	$Z = 6.19$ $p < .001$ $r = .61$	.729	$Z = 11.92$ $p < .001$ $r = .61$	.534	$Z = 12.16$ $p < .001$ $r = .61$
FCz	.000	$Z = 3.42$ $p < .001$ $r = .46$	.292	$Z = 6.20$ $p < .001$ $r = .61$	.315	$Z = 10.29$ $p < .001$ $r = .53$	.304	$Z = 12.12$ $p < .001$ $r = .61$
Fz	.182	$Z = 4.19$ $p < .001$ $r = .56$	.258	$Z = 6.17$ $p < .001$ $r = .61$	.000	$Z = 9.09$ $p < .001$ $r = .47$	.000	$Z = 3.46$ $p < .01$ $r = .17$

'Between groups' comparison for selected intervals of the corresponding limit ERP curves at 95% confidence is assessed by the paired Wilcoxon signed rank test and the statistics Z and effect size r are reported.
