# Supplementary material for: Event-Related Potentials during a Gambling Task in Young Adults with Attention-Deficit/Hyperactivity Disorder
Source: Front Hum Neurosci. 2018 Feb 27;12:79. doi: 10.3389/fnhum.2018.00079 (PMC5835343; doi:10.3389/fnhum.2018.00079)
Supplement: Supplementary file 4 [file Image1.pdf]

Mesrobian SK, Villa AEP, Bader M, Götte L and Lintas A (2018) Event-Related Potentials during a Gambling Task in Young Adults with Attention-Deficit/Hyperactivity Disorder. Front. Hum. Neurosci. 12:79. doi: 10.3389/fnhum.2018.00079

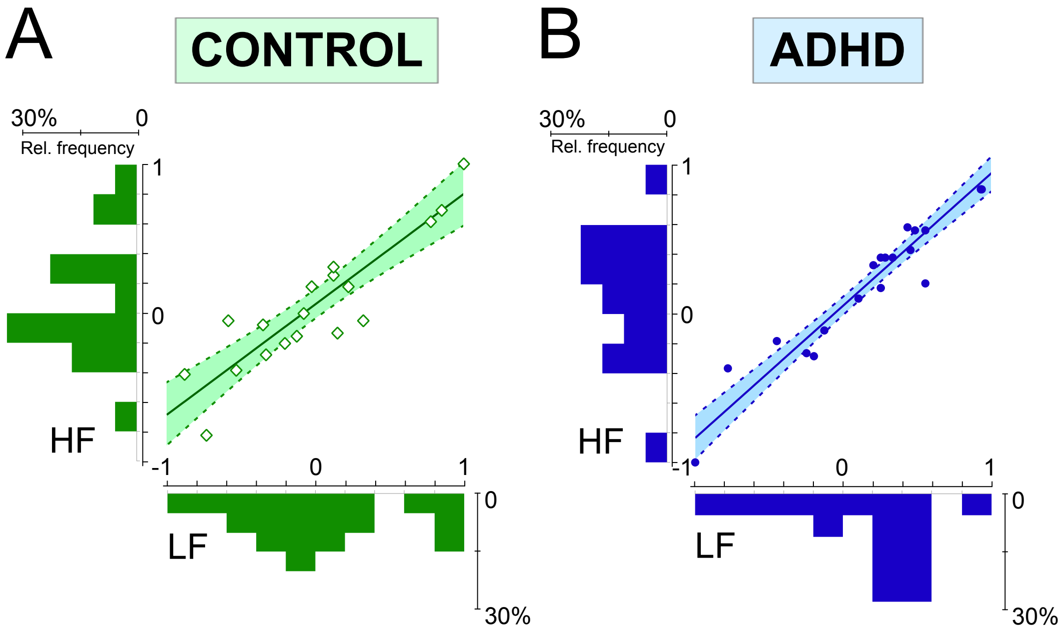

Figure S1. Individual strategies during outcome frequency feedback conditions. **A.** Control participants. Scatter plot of the risk index during high frequency feedback, RI(*HF*), as a function of the risk index during low frequency feedbackRI(*LF*). The robust regression equation is $y = 0.075 + 0.728x$ (F(1,16)=61.18, $p < .001$, $R^2 = .796$). **B.** Same scatter plot for ADHD patients, The robust regression equation is $y = 0.035 + 0.875x$ (F(1,16)=142.39, $p < .001$, $R^2 = .906$). Each point represents the data from one participant. Dashed lines represents 95% confidence interval. Histograms represents the marginal distributions of RI.
